# Investigating the use of finerenone in children with chronic kidney disease and proteinuria: design of the FIONA and open-label extension studies

**DOI:** 10.1186/s13063-024-08021-z

**Published:** 2024-03-21

**Authors:** Franz Schaefer, Giovanni Montini, Hee Gyung Kang, Johan Vande Walle, Joshua Zaritsky, Michiel F. Schreuder, Mieczyslaw Litwin, Andrea Scalise, Helen Scott, James Potts, Pablo Iveli, Stefanie Breitenstein, Bradley A. Warady

**Affiliations:** 1 Pediatric Nephrology Division, Heidelberg University Hospital, Heidelberg, Germany; 2 Department of Clinical Sciences and Community Health, University of Milan, Milan, Italy; 3 Department of Pediatric Nephrology, Dialysis and Transplant Unit, Fondazione IRCCS Ca’ Granda, Ospedale Maggiore Policlinico, Milan, Italy; 4 Department of Pediatrics, Seoul National University College of Medicine, Seoul, South Korea; 5 Department of Pediatric Nephrology, Ghent University Hospital, Erknet Center, C4C Ghent, Belgium; 6 Department of Nephrology, Phoenix Children’s Hospital, Phoenix, AZ USA; 7 Department of Pediatric Nephrology, Radboudumc Amalia Children’s Hospital, Nijmegen, The Netherlands; 8 Department of Nephrology and Arterial Hypertension, Children’s Memorial Health Institute, Warsaw, Poland; 9 Bayer Hispania, S.L, Sant Joan Despi, Barcelona, Spain; 10 Bayer U.S Pharmaceuticals, Whippany, NJ USA; 11 Bayer AG, Wuppertal, Germany; 12 Department of Pediatrics, University of Missouri–Kansas City School of Medicine, Kansas City, MO USA; 13 Children’s Mercy Kansas City, Kansas City, MO USA

**Keywords:** Chronic kidney disease, Finerenone, Mineralocorticoid, Pediatric, Proteinuria, Renoprotective therapy

## Abstract

**Introduction:**

Proteinuria is a modifiable risk factor for chronic kidney disease (CKD) progression in children. Finerenone, a selective, non-steroidal, mineralocorticoid receptor antagonist (MRA) has been approved to treat adults with CKD associated with type 2 diabetes mellitus (T2DM) following results from the phase III clinical trials FIDELIO-DKD (NCT02540993) and FIGARO-DKD (NCT02545049). In a pre-specified pooled analysis of both studies (*N* = 13,026), finerenone was shown to have an acceptable safety profile and was efficacious in decreasing the risk of adverse kidney and cardiovascular outcomes and of proteinuria.

**Objective:**

FIONA and the associated open-label extension (OLE) study aim to demonstrate that combining finerenone with an angiotensin-converting enzyme inhibitor (ACEi) or angiotensin receptor blocker (ARB) is safe, well-tolerated, and effective in sustainably reducing urinary protein excretion in children with CKD and proteinuria.

**Design:**

FIONA (NCT05196035; Eudra-CT: 2021–002071-19) is a randomized (2:1), double-blind, placebo-controlled, multicenter, phase III study of 6 months’ duration in approximately 219 pediatric patients. Patients must have a clinical diagnosis of CKD (an eGFR ≥ 30 mL/min/1.73 m^2^ if ≥ 1 to < 18 years or a serum creatinine level ≤ 0.40 mg/dL for infants 6 months to < 1 year) with significant proteinuria despite ACEi or ARB usage. The primary objective is to demonstrate that finerenone, added to an ACEi or ARB, is superior to placebo in reducing urinary protein excretion. FIONA OLE (NCT05457283; Eudra-CT: 2021–002905-89) is a single-arm, open-label study, enrolling participants who have completed FIONA. The primary objective of FIONA OLE is to provide long-term safety data.

FIONA has two primary endpoints: urinary protein-to-creatinine ratio (UPCR) reduction of ≥ 30% from baseline to day 180 and percent change in UPCR from baseline to day 180. A sample size of 198 participants (aged 2 to < 18 years) in FIONA will provide at least 80% power to reject the null hypothesis of either of the two primary endpoints.

**Conclusion:**

FIONA is evaluating the use of finerenone in children with CKD and proteinuria. Should safety, tolerability, and efficacy be demonstrated, finerenone could become a useful additional therapeutic agent in managing proteinuria and improving kidney outcomes in children with CKD.

**Trial registration:**

ClinicalTrials.gov NCT05196035. Registered on 19 January 2022.

## Plain language summary

High levels of protein in the urine are a common symptom in children with kidney disease and can lead to progressive kidney damage. The increased level of protein in the urine is a risk factor that can be targeted by drugs; therefore, drugs that can reduce protein in the urine may help protect the kidneys of children with kidney disease from disease progression.

Finerenone is a drug that is used to protect the heart and kidneys of adults with kidney disease and type 2 diabetes. Based on its effects in adults, finerenone is currently being studied in children in the FIONA and FIONA OLE studies. The FIONA and FIONA OLE studies aim to find out whether adding finerenone to current standard drugs is well-tolerated and effective in reducing the level of protein in the urine of children with kidney disease.

Here, the authors describe the rationale and design of the FIONA and FIONA OLE studies. Treatment in FIONA will last approximately 6 months and will include children and adolescents aged from 6 months to < 18 years old. Children who have completed the FIONA study will be given the opportunity to enroll onto the FIONA OLE study, which will evaluate long-term safety. If finerenone is found to be effective and have tolerable side effects, it could become a useful additional drug to manage protein levels in the urine and protect the kidneys of children with kidney disease.

## Introduction

National epidemiological studies in Asian, European, and North and South American countries suggest that the prevalence of CKD in children is 30–100 per million age-related population per year [1]. The most common causes of CKD in children include congenital anomalies of the kidneys and urinary tract (CAKUT) followed by glomerular and systemic immunological diseases [2]. Children with CKD are at risk for increased morbidity and mortality and decreased quality of life, and a significant percentage of children with CKD will develop kidney failure by 20 years of age [3, 4]. Most importantly, the incidence and prevalence of all stages of CKD in children continues to increase worldwide [3, 5].

To date, only renin–angiotensin–aldosterone system (RAAS) blockade with an angiotensin-converting enzyme inhibitor (ACEi) or angiotensin receptor blocker (ARB) has been shown to reduce proteinuria and improve kidney outcomes in children [6–10]. However, despite treatment with RAAS blockade therapy, many patients with CKD continue to have persistent proteinuria and progression of kidney disease [6–10]. Therefore, novel treatment options are required to target modifiable risk factors, complement current therapies, and improve outcomes in children with CKD.

Aldosterone-mediated activation of the mineralocorticoid receptor (MR) in the kidney promotes tissue inflammation and injury; this manifests as glomerulosclerosis and proteinuria, driving CKD progression [11, 12]. The use of finerenone in various CKD rodent models reduced fibrosis and markers of oxidative stress and inflammation in the kidney and endothelium, with improved endothelial function and reduced proteinuria [12]. Finerenone, a selective, non-steroidal MR antagonist (MRA), is approved in the USA, European Union, and several other countries, including Japan and the UK, for the treatment of adults with CKD associated with type 2 diabetes mellitus (T2DM) to reduce the risk of adverse kidney and cardiovascular outcomes [13–15]. Approval followed positive results being generated from the phase III FIDELIO-DKD (NCT02540993) and FIGARO-DKD (NCT02545049) studies [16–18].

The FIDELITY study is a prespecified pooled analysis of the FIDELIO-DKD and FIGARO-DKD studies (both FIDELIO-DKD and FIGARO-DKD excluded patients with an eGFR < 25 mL/min/1.73 m^2^), forming the largest heart and kidney outcomes program in patients with CKD and T2DM to date. In this pooled analysis (*N* = 13,026), finerenone in addition to maximum RAAS inhibition reduced the risk of the composite cardiovascular outcome (cardiovascular death, non-fatal myocardial infarction, non-fatal stroke, or hospitalization for heart failure) by 14% compared with placebo. Similarly, finerenone reduced the risk of the composite kidney outcome (kidney failure, a sustained ≥ 57% decrease in estimated glomerular filtration rate from baseline over ≥ 4 weeks, or renal death) by 23% compared with placebo [18]. Based on results in adults with CKD and T2DM, it is anticipated that combining finerenone with an ACEi or ARB will exert comparable beneficial effects on urinary protein excretion and kidney function in adults with CKD without T2DM, and in children with CKD and proteinuria, potentially providing a novel therapeutic strategy for the pediatric population [19].

The aim of the FIONA study is to demonstrate that finerenone, in addition to an ACEi or ARB, is superior to placebo plus an ACEi or ARB in reducing urine protein excretion in children with CKD and proteinuria. Data on the long-term safety of finerenone use in children with CKD is being obtained from the FIONA open-label extension (OLE) study. The design of the FIONA and FIONA OLE studies are presented here.

## Materials and methods

### FIONA study design, organization, and support

FIONA (NCT05196035) is an ongoing 6-month, multicenter, randomized, double-blind, placebo-controlled phase III study. It is being conducted in around 100 sites across approximately 25 countries. Screening for participation began in March 2022, and the estimated completion date is March 2027. Approximately 219 participants will be randomly assigned to the study interventions.

The FIONA program was designed with input from representatives of pediatric kidney disease networks (European Study Consortium for CKD Affecting Pediatric Patients (ESCAPE) and North American Pediatric Renal Trials and Collaborative Studies (NAPRTCS)) [20] as well as from Conect4Children (c4c), a large collaborative European network that aims to facilitate the development of new drugs and other therapies in pediatric populations. ESCAPE, NAPRTCS, and c4c support the operational conduct of the trial [20–22]. Moreover, c4c and the Institution for Advanced Clinical Trials (I-ACT) have facilitated patient, parent, and ethics input into the trial design and discussion of endpoints [23].

**Fig. 1:**
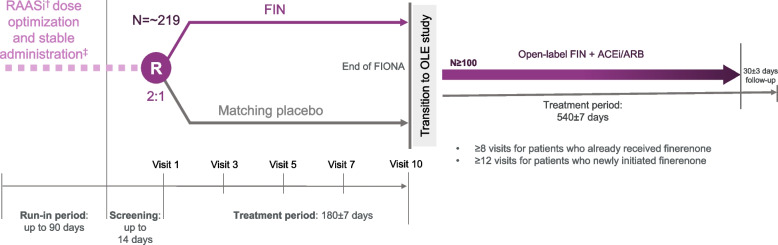
Study design. Dagger indicates RAASi = ACEi or ARB. Double dagger indicates mandatory for at least 30 days before screening. ACEi angiotensin-converting enzyme inhibitor, ARB angiotensin receptor blocker, EOT end of treatment, FIN finerenone, OLE open-label extension, RAASi renin–angiotensin–aldosterone system inhibitor

The trial includes a run-in phase of up to 90 days prior to screening. Initiation and/or dose optimization of ACEi or ARB therapy will occur in the first 60 days, followed by a period of at least 30 days on a stable optimized dose of ACEi or ARB prior to screening. A screening visit will take place ≤ 14 days prior to randomization. Following randomization, subsequent visits will take place at 30, 60, 90, and 180 days, as well as 3–7 and 30 days after study drug initiation and up-titration. A safety follow-up visit will be scheduled 30 days after the final study drug dose (day 210) for any participants who do not transition to the OLE study (Fig. 1).

### FIONA: study participants

Eligible participants will be aged 6 months to < 18 years, with a clinical diagnosis of CKD (an eGFR ≥ 30 mL/min/1.73 m^2^ if aged ≥ 1 to < 18 years of age or a serum creatinine level ≤ 0.40 mg/dL for infants aged 6 months to < 1 year) and clinically relevant proteinuria despite ACEi or ARB usage at screening. Key eligibility criteria are detailed in Table 1.

**Table 1:** FIONA key eligibility criteria

Inclusion criteria	Exclusion criteria
Age 6 months to < 18 years	Planned urological surgery expected to influence kidney function or scheduled renal transplant within the study time frame
	Systemic hypertension (stage 2 as defined by the institutional guidelines on BP management
	Systemic hypotension defined as symptomatic hypotension or a mean systolic BP below the 5th percentile for age, sex, and height but no lower than 80 mmHg for participants < 18 years and symptomatic hypotension or a mean SBP < 90 mmHg in participants ≥ 18 years at visit 1
CKD stages 1–3^a^ (eGFR ≥ 30 mL/min/1.73 m^2^) for children aged ≥ 1 year to < 18 years or serum creatinine^b^ ≤ 0.40 mg/dL for infants aged 6 months to < 1 year	Children with hemolytic uremic syndrome diagnosed ≤ 6 months prior to screening
	Participants on high dose glucocorticoids, cyclophosphamide, or biological therapies (rituximab and abatacept) within < 6 months prior to screening
Proteinuria defined as UPCR of ≥ 0.50 g/g in patients aged ≥ 2 years with CKD stage 2 or 3, or UPCR ≥ 1.0 g/g for patients aged < 2 years or ≥ 2 years with CKD stage 1	Patients with nephrotic syndrome receiving albumin infusions or with acute kidney injury requiring dialysis within the last 6 months prior to screening
On a maximum tolerated dose of RAASi	Concomitant therapy with an MRA, renin inhibitor, SGLT2i, ARNI, or potassium-sparing diuretic within 30 days prior to screening
Serum [K^+^] ≤ 5.0 mmol/L for children ≥ 2 years old and ≤ 5.3 mmol/L for children < 2 years old	Concomitant therapy with both ACEi and ARBs together
	Concomitant therapy with strong CYP3A4 inhibitors, or moderate or strong CYP3A4 inducers within 7 days prior to randomization

*ACEi* Angiotensin-converting enzyme inhibitor, *ARB* Angiotensin receptor blocker, *ARNI* Angiotensin receptor neprilysin inhibitor, *BP* Blood pressure, *CKD* Chronic kidney disease, *CYP3A4* cytochrome P450 3A4, *eGFR* estimated glomerular filtration rate, *IV* Intravenous, *[K*^+^*]* Potassium, *MRA* Mineralocorticoid receptor antagonist, *RAASi* Renin–angiotensin–aldosterone system inhibitor, *SGLT2i* Sodium/glucose cotransporter-2 inhibitor, *UPCR* Urinary protein-to-creatinine ratio

^a^CKD stage 1 defined as eGFR ≥ 90 mL/min/1.73 m^2^; CKD stage 2 defined as eGFR ≥ 60 to < 90 mL/min/1.73 m^2^; and CKD stage 3 defined as eGFR ≥ 30 to < 60 mL/min/1.73 m^2^. Patients must have a stable kidney function between screening and baseline, defined as no increase or decrease in eGFR ≥ 15% for children ≥ 1 to > 18 years of age

^b^Patients must have a stable kidney function between screening and baseline, defined as no increase or decrease in creatinine ≥ 0.10 mg/dL for children aged < 1 year

The planned study will follow an age-staggered approach, with adolescents aged ≥ 12 years enrolled first. Four age groups are planned (12 to < 18 years, ≥ 6 to < 12 years, ≥ 2 to < 6 years, and ≥ 6 months to < 2 years). At the time of submission of this publication, children and adolescents aged 6 years to < 18 years are being enrolled to this study. Prior to initiating enrollment of the subsequent younger age cohort, an assessment of safety, tolerability, and pharmacokinetics (PK) will be performed by an independent external data monitoring committee (DMC) when approximately two-thirds of the minimum number of participants in the previous older age cohort have completed 90 days of treatment.

### FIONA: randomization and study treatment

Participants will be randomized 2:1 to finerenone plus standard of care or to placebo plus standard of care (Fig. 1). Randomization will be stratified according to CKD etiology (glomerular vs non-glomerular disease, defined by the investigator) and urinary protein-to-creatinine ratio (UPCR) category (average screening UPCR < 1.0 g/g vs ≥ 1.0 g/g). Participants will receive finerenone or placebo in addition to their standard of care background treatment with an ACEi or ARB (according to guidelines on blood-pressure management), other antihypertensive treatment (as needed), and immunosuppression (at stable doses for ≥ 90 days, if applicable). The study drug will be administered once daily.

### FIONA: study objectives and endpoints

The primary objective of the study will be to demonstrate that finerenone, in addition to an ACEi or ARB, is superior to placebo combined with an ACEi or ARB in reducing urine protein excretion. There will be two alternative primary endpoints to address this objective:

1. UPCR reduction of at least 30% from baseline to day 180
2. Percent change in UPCR from baseline to day 180

The secondary objectives, as detailed in Table 2, are to assess the safety profile and to provide further evidence for the efficacy of finerenone compared with placebo in addition to standard of care in children. The systemic exposure of finerenone and the pediatric formulation will also be assessed.

**Table 2:** FIONA study objectives and endpoints

Objectives	Endpoints
FIONA	
Primary	
• To demonstrate that finerenone in addition to an ACEi or ARB is superior to placebo in reducing urinary protein excretion	• UPCR reduction of at least 30% from baseline to day 180 ± 7^a^
	• Percent change from baseline in UPCR to day 180 ± 7^a^
Secondary	
• To assess the safety profile of finerenone in addition to SoC in children with CKD compared with placebo	• Number of participants with TEAEs
	• Change in serum K^+^, serum creatinine, eGFR, and SBP from baseline to day 180 ± 7
• To further support the efficacy of finerenone in addition to SoC in children with CKD	
	• UPCR reduction of at least 30% from baseline to day 180 ± 7^a^
	• Percent change in UPCR from baseline to day 180 ± 7^a^
• To confirm the dose and systemic exposure of finerenone in children with CKD	
	• Change in UACR from baseline to day 180 ± 7
• To assess the acceptability and palatability of the pediatric formulation	• PK (finerenone *C*_max,md_, AUC_t,md_) based on total concentrations in plasma
	• Taste and texture of the pediatric formulation

*ACEi* Angiotensin-converting enzyme inhibitors, *ARB* Angiotensin receptor blocker, *AUC*_*t,md*_ Area under the curve for time after multiple doses, *CKD* Chronic kidney disease, *C*_*max,md*_ Maximum observed drug concentration after multiple doses, *eGFR* estimated glomerular filtration rate, *K*^+^ Potassium, *PK* Pharmacokinetics, *SBP* Systolic blood pressure, *SoC* Standard of care, *TEAE* Treatment-emergent adverse event, *UACR* Urinary albumin-to-creatinine ratio, *UPCR* Urinary protein-to-creatinine ratio

^a^These two primary endpoints are not considered as co-primary endpoints and the respective other endpoint is considered as a secondary endpoint

### FIONA: assessments

Demographic characteristics will be recorded during the run-in phase. Medical history and other pertinent clinical information will be recorded at screening and at visit 1. For the run-in visit, UPCR will be determined from a single urine sample collected locally. For screening and all subsequent scheduled visits, UPCR will be determined from the average of three first morning urine samples collected on consecutive days by the central laboratory. Estimated glomerular filtration rate (eGFR) will be calculated at least monthly during the first 3 months and at the end of the study using a central laboratory serum creatinine measurement, except in participants for whom this would exceed the allowed daily/monthly blood sampling volume, in which case serum creatinine and eGFR will be assessed locally only. The Chronic Kidney Disease in Children (CKiD) U25 equation will be used to calculate central eGFR [24].

Safety will be assessed at all scheduled visits by recording the frequency and severity of adverse events (AEs). At all scheduled visits, serum potassium and creatinine will be monitored for safety assessments and blood pressure will be measured using an automated oscillometric device. The average of three blood pressure readings will be recorded in the eCRF. A 12-lead electrocardiogram will be recorded at visit 1 (baseline), visit 3, and visit 10. Echocardiography will also be performed at visit 1 (baseline) and at visit 10 or premature discontinuation to explore potential cardiovascular comorbidity and treatment effects. Health-related quality of life will be assessed using the Pediatric Quality of Life Inventory™ questionnaire at visit 1 (baseline) and visit 10.

### FIONA: statistical considerations

#### Sample size assumptions

##### UPCR reduction of at least 30% from baseline to day 180

It is expected that sample sizes of 198 participants, aged ≥ 2 to < 18 years, respectively, will achieve at least 80% power to detect a difference in responder rates of ≥ 20% between finerenone and placebo. FIONA will be powered for a smaller treatment effect than that previously observed in adults with T2DM and CKD to account for the possibility that the difference in responder rates may be smaller in the pediatric population. Should the difference in responder rates be ≥ 25% for ≥ 30% reduction from baseline in UPCR, a sample size of 198 will yield a statistical power of > 90%.

##### Percent change in UPCR from baseline to day 180

A sample size of 198 participants, aged ≥ 2 to < 18 years old, will also achieve at least 80% power to detect a difference of 31.6% in mean UPCR reduction from baseline with finerenone compared with placebo. A log-normal distribution for the UPCR and a standard deviation of the difference between the log-transformed UPCR values of 0.95 is assumed. Assumptions for the UPCR ratios from baseline to day 180 and the corresponding standard deviation are based on a study in children with CKD and proteinuria receiving an ARB [10], as well as the phase IIb study ARTS-DN [25] and the phase III study FIDELIO-DKD [16] which investigated finerenone usage in adults.

### FIONA: statistical analysis

#### Efficacy

A subset of the full analysis set (SFAS), including participants ≥ 2 years to < 18 years of age will be used for the primary outcomes analyses. All other efficacy variables will be analyzed using the full analysis set (FAS), defined as all randomized participants.

To determine whether finerenone is superior to placebo in the proportion of responders at day 180, a Pearson’s χ2 test with a one-sided 5% significance level will be used where response is defined as a ≥ 30% reduction in UPCR compared with baseline. Missing UPCR data will be imputed using multiple imputation based on a missing at random assumption; if the baseline value is missing, the screening value will be used. An analysis of covariance, adjusted for baseline log_(UPCR)_ (continuous) and glomerular disease at baseline, will be used to estimate the difference in percentage change in UPCR from baseline to day 180 between participants receiving finerenone and those receiving placebo. The least squares mean difference between the treatment groups at day 180 will be used to evaluate the treatment effect of percent change from baseline UPCR.

#### Safety

The safety analysis set (SAF), defined as all randomized participants who have taken at least one dose of study medication, will be used to summarize the safety variables. All treatment-emergent AEs will be summarized overall and by treatment group in the SAF population.

### FIONA OLE: study design

**Fig. 2:**
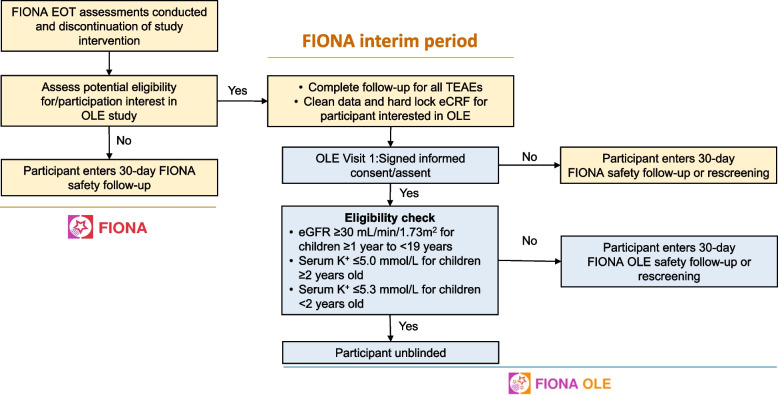
Transition from FIONA to FIONA OLE study. Dagger indicates the key list of inclusion and exclusion criteria that must be fulfilled can be found in Table 1 and 2. eCRF electronic case report form, eGFR estimated glomerular filtration rate, EOT end of treatment, K + potassium, OLE open-label extension, TEAE treatment-emergent adverse event

The FIONA OLE (NCT05457283) study is an 18-month, multicenter, single-arm, open-label study (Fig. 1). Participants who are not eligible to participate in FIONA OLE at the FIONA end-of-treatment (EoT) visit (e.g., eGFR below threshold for inclusion) or who do not want to enroll into the OLE, will enter the 30-day safety follow-up period of FIONA. Participants who are willing and likely to be eligible to transition from FIONA to FIONA OLE will enter an interim period of 7 days (Fig. 2). Following this, the FIONA electronic case report form will be locked to allow for unblinding of eligible participants in the OLE. Once participants have signed the informed consent or assent form for FIONA OLE, the eligibility criteria will be checked (Table 3). Eligible participants will be unblinded at OLE visit 1 regarding their previous treatment assignment to allow them to make an informed decision to continue in the OLE trial. Ineligible participants or those choosing not to participate in the OLE trial after unblinding will continue to receive standard of care according to their physician’s recommendation and will enter the 30-day safety follow-up period of FIONA OLE. The treatment duration will be up to 540 (± 7) days per participant, with ≥ 8 visits planned for participants who had already received finerenone and ≥ 12 visits for participants newly receiving finerenone.

**Table 3:** FIONA OLE key eligibility criteria

Inclusion criteria	Exclusion criteria
Age ≥ 1 to < 19 years	Planned urological surgery expected to influence renal function
Prior participation in FIONA study	Patients who are candidates for renal transplantation
CKD stages 1–3 (eGFR ≥ 30 mL/min/1.73 m^2^) for children aged ≥ 1 year to < 19 years at FIONA EoT and visit 1	Systemic hypertension (stage 2 as defined by the institutional guidelines on BP management
On a maximum tolerated dose of RAASi	Systemic hypotension defined as symptomatic hypotension or a mean systolic BP below the 5th percentile for age, sex, and height but no lower than 80 mmHg for participants < 18 years and symptomatic hypotension or a mean SBP < 90 mmHg in participants ≥ 18 years at visit 1
Serum K^+^ ≤ 5.0 mmol/L for children ≥ 2 years old and ≤ 5.3 mmol/L for children < 2 years old at both FIONA EoT and visit 1	Concomitant therapy with an MRA, any renin inhibitor, any SGLT2i, ARNI or potassium-sparing diuretic
	Concomitant therapy with both ACEi and ARBs together
	Concomitant therapy with strong CYP3A4 inhibitors, moderate or strong CYP3A4 inducers

*ACEi* Angiotensin-converting enzyme inhibitor, *ARB* Angiotensin receptor blocker, *ARNI* Angiotensin receptor neprilysin inhibitor, *BP* Blood pressure, *CKD* Chronic kidney disease, *CYP3A4* Cytochrome P450 3A4, *eGFR* estimated glomerular filtration rate, *EoT* End of treatment, *K*^+^ Potassium, *MRA* Mineralocorticoid receptor antagonist, *OLE* Open label extension, *RAASi* Renin–angiotensin–aldosterone system inhibitor, *SBP* Systolic blood pressure, *SGLT2i* Sodium/glucose cotransporter-2 inhibitor

### FIONA OLE: study treatments

Participants who have previously received finerenone in FIONA will remain on the same dose they had been receiving at EoT unless a dose modification is warranted.

Participants who have received placebo in FIONA will switch to finerenone in FIONA OLE.

### FIONA OLE: study objectives and endpoints

The primary objective of the FIONA OLE study will be to provide long-term safety data regarding the use of finerenone in addition to current standard of care (an ACEi or ARB). There will be three primary endpoints to address this objective:

1. Number of participants with treatment-emergent adverse events (TEAEs)
2. Change in serum potassium levels from baseline to day 540
3. Change in systolic blood pressure (SBP) from baseline to day 540.

The secondary and other exploratory endpoints, as detailed in Table 4, are to assess the long-term effects of finerenone on proteinuria and kidney function.

**Table 4:** FIONA OLE study objectives and endpoints

Objectives	Endpoints
FIONA OLE	
Primary	
• To demonstrate that finerenone in addition to an ACEi or ARB is safe when given long term	• Number of participants with TEAEs
	• Change in serum K^+^ levels from baseline to day 540 ± 7
	• Change in SBP from baseline to day 540 ± 7
Secondary	
• To assess the long-term treatment effects of finerenone in addition to SoC on proteinuria and kidney function	• Change in UPCR and UACR from baseline to day 540 ± 7
	• Change in eGFR from baseline to day 540 ± 7

*ACEi* Angiotensin-converting enzyme inhibitors, *ARB* Angiotensin receptor blocker, *eGFR* estimated glomerular filtration rate, *K*^+^ Potassium, *SBP* Systolic blood pressure, *SoC* Standard of care, *TEAE* Treatment-emergent adverse event, *UACR* Urinary albumin-to-creatinine ratio, *UPCR* Urinary protein-to-creatinine ratio

### FIONA OLE: assessments

Medical history will be recorded at visit 1 of the FIONA OLE study. There will be at least quarterly visits with UPCR determined from the average of three first morning urine samples taken on consecutive days and eGFR calculated. Participants who have newly started finerenone will have monthly visits in the first 3 months for safety assessments.

Blood pressure will be measured at all scheduled visits, using an automated oscillometric device. The average of three blood pressure readings will be recorded in the eCRF. Serum potassium will be monitored for safety assessments at all scheduled visits and determined both centrally and locally as part of the limited chemistry assessments. A 12-lead electrocardiogram will be recorded at visits 10 and 14 for all patients. Echocardiography will be performed approximately every 6 months. Visits to assess safety and tolerability of the study drug are scheduled 3–7 and 30 days after newly starting finerenone. Review of AEs and serious adverse events will be ongoing at all visits. Health-related quality of life will be assessed using the Pediatric Quality of Life Inventory™ questionnaire at visit 10, visit 12, and EoT.

### FIONA OLE: statistical considerations

Statistical methods for the OLE study will be descriptive and exploratory in nature, and no formal statistical hypotheses are planned. There is also no formal sample size calculation for this study. The SAF, including participants who complete the FIONA study and meet the OLE eligibility criteria, will be used to summarize safety variables. The secondary UPCR and urinary albumin to creatinine ratio (UACR) efficacy analyses will be conducted in the FAS and the SFAS; the latter will exclude participants < 2 years old.

### Study oversight

The study protocol, any protocol amendments, and informed consent and assent forms are subject to approval following review by institutional review boards and independent ethics committees. FIONA and the associated OLE study are being conducted in compliance with the principles of the Declaration of Helsinki and in accordance with the International Conference on Harmonization Guidelines for Good Clinical Practice. All study participants who have reached the legal age will provide informed consent; participants younger than the legal age of consent will provide informed assent if possible. Parent(s) or guardian(s) will also be asked to provide written consent for participants younger than the legal age of consent. The studies are registered with www.clinicaltrials.gov (NCT05196035 and NCT05457283).

The studies are being overseen by a steering committee, composed of external experts in pediatric nephrology, to ensure the overarching integrity of the study. The sponsor is responsible for the collection and analysis of data in conjunction with the authors. The FIONA and associated OLE study are funded by Bayer AG. An independent data- and safety-monitoring committee will be reviewing safety and exposure data and overall study conduct throughout the trial.

## Discussion

FIONA is the first phase III study investigating the efficacy and safety of finerenone in addition to standard of care in children with CKD and proteinuria. Despite treatment with ACEi or ARBs, children with CKD continue to have residual proteinuria and kidney disease progression. Sodium/glucose cotransporter-2 inhibitors (SGLT2is) have not been studied in children with CKD, and concomitant treatment with an SGLT2i in FIONA and the OLE is excluded. FIONA will assess the clinical benefits of combining finerenone with standard of care in reducing urinary protein excretion, using UPCR reduction of at least 30% and percent change in UPCR as the two alternative primary endpoints.

Proteinuria is an important modifiable risk factor for CKD progression in both children and adults with CKD. As proteinuria has been shown to have an important role in the pathophysiology of kidney disease progression in adults and children, an early change in proteinuria may be a valid surrogate endpoint [7, 26]. In pediatric CKD, proteinuria may result from increased excretion of albumin (as is common in adult and pediatric patients with glomerular disease) or non-albumin proteins due to tubulointerstitial diseases (which are more common in pediatric CKD) or a combination of the two. Therefore, the Kidney Disease Improvement Global Outcomes (KDIGO) guidelines recommend the use of UPCR rather than UACR for measuring protein excretion in children with CKD [27, 28]. In an analysis of the pooled CKiD and ESCAPE studies, which included both children with glomerular and non-glomerular disease, empirical incidence rates for the composite kidney outcome (defined as the earliest of either a 50% reduction of baseline GFR, or eGFR < 15 mL/min/1.73 m^2^, or kidney failure) increased substantially from 1.5 to 8.1 per 100 person-years in those with CKD stage 2 and almost doubled in CKD stages 3a and 3b for patients with UPCR between 0.5 and 2 mg/mg compared with those with a UPCR of < 0.5 mg/mg [29]. Furthermore, a longitudinal analysis of the CKiD data demonstrated the predictive effect of baseline proteinuria on time-to-event analyses [30]. UPCR > 0.5 g/g is considered severely increased according to KDIGO; therefore, this threshold was chosen to define eligibility for children aged ≥ 2 years in this study [28]. For children aged < 2 years and for all children with CKD stage 1, a UPCR threshold of ≥ 1 g/g was used to enrich the trial with a high-risk population. Similar prognostic abilities of UPCR, UACR, and urine non-albumin to creatinine ratio to predict an associated increased risk for a > 50% decline in eGFR or kidney failure have been demonstrated in additional pediatric CKD studies [31]. These analyses highlight the appropriateness of reducing proteinuria in children with CKD.

Analyses from previous trials suggest that a reduction of proteinuria of ≥ 30% is associated with improved kidney outcomes in both adults and children with CKD, with reductions of up to approximately 45% seen in some trials [7, 10, 32, 33]. Secondary analysis of the ESCAPE trial demonstrated that a larger initial reduction in proteinuria was associated with a greater risk reduction for the primary composite kidney endpoint (sustained 50% reduction of eGFR or progression to kidney failure) in children. The analysis from the ESCAPE trial demonstrated that the subgroup with the greatest initial proteinuria reduction (> 60%) experienced the lowest risk for the composite kidney outcome (hazard ratio 0.42; 95% confidence interval 0.22–0.79) [7]. While the FIONA study is open for the enrolment of children with CKD caused by both glomerular and non-glomerular diseases, the inclusion criteria are expected to lead to the preferential selection of patients with immune-mediated glomerular diseases and treatment-refractory proteinuria in early CKD stages. This population is at significant risk of kidney function decline and is likely to benefit from preservation of eGFR at early CKD stages [34]. However, it is expected that with younger age, there will be an increasing proportion of patients with non-glomerular disease, and both the ESCAPE study and the study by Webb et al. [10] showed similar treatment effects on proteinuria, irrespective of the CKD etiology.

Results from FIDELITY indicate that finerenone delays kidney disease progression in patients who are already receiving treatments known to reduce protein excretion. In the pooled FIDELITY analysis, finerenone reduced mean UACR by 32% from baseline to 4 months compared with placebo [35]. Based on its mode of action and the consistent benefit seen in related populations, finerenone is anticipated to demonstrate comparable efficacy in pediatric patients with CKD and proteinuria. As expected by the mode of action of an MRA, there was an increased risk of hyperkalemia associated with the use of finerenone in FIDELITY; however, the majority of these events were non-serious and the number of events leading to hospitalizations was low [35]. The analysis demonstrates that, in adults, hyperkalemia is manageable using a serum potassium-guided dose regimen. In the FIDELIO-DKD trial, patients in the finerenone and placebo groups had a similar risk of acute kidney injury (AKI)-related AEs [16]. This is particularly important in the pediatric population, as acute illness-related dehydration can be more common in children than adults [36]. There remains a need to obtain further information about the impact of combining RAAS blockade with finerenone on the potential risk of AKI due to acute illness-related dehydration.

Selection of UPCR, a surrogate marker for kidney damage, as the primary efficacy outcome for FIONA, enables the study to have sufficient statistical power with relatively short follow-up and inclusion of a feasible number of pediatric participants. Assuming the treatment effects on eGFR in children with CKD are similar to those observed in adult CKD studies, selection of the eGFR slope as a surrogate endpoint would require a large number of participants and a follow-up of at least 2 years, as demonstrated by respective adult trial analyses and simulations [37–40]. Such a study duration was not considered acceptable for a placebo-controlled trial, given the vulnerable pediatric population and the expected positive benefit-to-risk profile of finerenone weighed against the substantial patient and family burden associated with the number of study visits and procedures. Most importantly, families indicated acceptance of a proteinuria endpoint and the preference of a shorter study duration, which would also likely enhance subject participation. An event-driven study would require an even longer study duration and significantly more participants to accrue a sufficient number of events compared with a study investigating eGFR decline [38, 39] and therefore, UPCR as a surrogate endpoint was selected for this study.

A pooled analysis of the FIONA and FIONA OLE data is planned. In this pooled analysis, the change in UPCR, UACR, and eGFR from baseline in FIONA to EoT in FIONA OLE will be summarized. In addition, Bayesian modeling of the temporal dynamics of eGFR will be explored. The proposed disease progression model will assume a linear or piecewise linear decline of the eGFR over time, where intercept and slopes are modeled using population-level and participant-level effects.

In summary, FIONA is the first randomized controlled trial to investigate the efficacy and safety of finerenone combined with standard of care, specifically ACEi or ARB therapy, in children with CKD and proteinuria. Should a greater reduction in urinary protein excretion associated with finerenone compared with placebo be established, it can be inferred from the adult dataset that early intervention with finerenone may reduce proteinuria and slow kidney disease progression in the pediatric CKD population. The innovative study program design allows patients to transition from the FIONA trial to the FIONA OLE trial without washout and in an informed manner and provides the additional benefit of a longer follow-up period. The proposed pooled analysis of FIONA and FIONA OLE will provide a more robust characterization of the efficacy and safety profile of finerenone and further contribute to the understanding of the benefit-to-risk profile of using finerenone in children with CKD.

## Data Availability

Availability of the data underlying this publication will be determined according to Bayer’s commitment to the EFPIA/PhRMA “Principles for responsible clinical trial data sharing”. This pertains to scope, timepoint, and process of data access. As such, Bayer commits to sharing upon request from qualified scientific and medical researchers patient-level clinical trial data, study-level clinical trial data, and protocols from clinical trials in patients for medicines and indications approved in the United States (US) and European Union (EU) as necessary for conducting legitimate research. This applies to data on new medicines and indications that have been approved by the EU and US regulatory agencies on or after January 01, 2014. Interested researchers can use www.vivli.org to request access to anonymized patient-level data and supporting documents from clinical studies to conduct further research that can help advance medical science or improve patient care. Information on the Bayer criteria for listing studies and other relevant information is provided in the member section of the portal. Data access will be granted to anonymized patient-level data, protocols, and clinical study reports after approval by an independent scientific review panel. Bayer is not involved in the decisions made by the independent review panel. Bayer will take all necessary measures to ensure that patient privacy is safeguarded.

## Abbreviations

AE: Adverse events
AKI: Acute kidney injury
ARB: Angiotensin receptor blocker
ACEi: Angiotensin-converting enzyme inhibitor
CKD: Chronic kidney disease
CKDid: Chronic Kidney Disease in Children
CAKUT: Congenital anomalies of the kidneys and urinary tract
DKD: Diabetic kidney disease
DMC: Data monitoring committee
eGFR: Estimated glomerular filtration rate
ESCAPE: European Study Consortium for CKD Affecting Pediatric Patients
EOT: End-of-treatment
FAS: Full analysis set
I-ACT: Institution for Advanced Clinical Trials
KDIGO: Kidney Disease Improvement Global Outcomes
MR: Mineralocorticoid receptor
MRA: MR antagonist
NAPRTCS: North American Pediatric Renal Trials and Collaborative Studies
OLE: Open-label extension
PK: Pharmacokinetics
RAAS: Renin–angiotensin–aldosterone system
SAF: Safety analysis set
SBP: Systolic blood pressure
SFAS: Subset of the full analysis set
T2DM: Type 2 diabetes mellitus
TEAEs: Treatment-emergent adverse events
UACR: Urinary albumin to creatinine ratio
UPCR: Urinary protein-to-creatinine ratio
