# Correction: Investigating the use of finerenone in children with chronic kidney disease and proteinuria: design of the FIONA and open-label extension studies

**DOI:** 10.1186/s13063-026-09525-6

**Published:** 2026-02-04

**Authors:** Franz Schaefer, Giovanni Montini, Hee Gyung Kang, Johan Vande Walle, Joshua Zaritsky, Michiel F. Schreuder, Mieczyslaw Litwin, Andrea Scalise, Helen Scott, James Potts, Pablo Iveli, Stefanie Breitenstein, Bradley A. Warady

**Affiliations:** 1 Pediatric Nephrology Division, Heidelberg University Hospital, Heidelberg, Germany; 2 Department of Clinical Sciences and Community Health, University of Milan, Milan, Italy; 3 Department of Pediatric Nephrology, Dialysis and Transplant Unit, Fondazione IRCCS Ca’ Granda, Ospedale Maggiore Policlinico, Milan, Italy; 4 Department of Pediatrics, Seoul National University College of Medicine, Seoul, South Korea; 5 Department of Pediatric Nephrology, Ghent University Hospital, Erknet Center, C4C Ghent, Belgium; 6 Department of Nephrology, Phoenix Children’s Hospital, Phoenix, AZ USA; 7 Department of Pediatric Nephrology, Radboudumc Amalia Children’s Hospital, Nijmegen, The Netherlands; 8 Department of Nephrology and Arterial Hypertension, Children’s Memorial Health Institute, Warsaw, Poland; 9 Bayer Hispania, S.L, Sant Joan Despi, Barcelona, Spain; 10 Bayer U.S Pharmaceuticals, Whippany, NJ USA; 11 Bayer AG, Wuppertal, Germany; 12 Department of Pediatrics, University of Missouri–Kansas City School of Medicine, Kansas City, MO USA; 13 Children’s Mercy Kansas City, Kansas City, MO USA

**Correction: Trials 25, 203 (2024)**

**https://doi.org/10.1186/s13063-024-08021-z**

Following the publication of the original article [1], we were notified that a potential competing interest regarding consulting fees received from Bayer was inadvertently omitted from his disclosures.

Originally published sentence: "BAW discloses consultant fees from Amgen, Roche, and GSK."

Corrected sentence: "BAW discloses consultant fees from Bayer, Amgen, Roche, and GSK."

The original article has been corrected.
